# Spatial distribution of under immunization among children 12–23 months old in Butajira HDSS, southern Ethiopia

**DOI:** 10.1186/s12887-021-02690-4

**Published:** 2021-05-10

**Authors:** Admassu Ketsela, Seifu Hagos Gebreyesus, Wakgari Deressa

**Affiliations:** 1 Menelik II Medical & Health Sciences College, Kotebe Metropolitan University, Addis Ababa, Ethiopia; 2 Department of Nutrition and Dietetics, School of Public Health, Addis Ababa University, Addis Ababa, Ethiopia; 3 Department of Preventive Medicine, School of Public Health, Addis Ababa University, Addis Ababa, Ethiopia

**Keywords:** Butajira HDSS, Cluster, Ethiopia, Spatial analysis and under immunization

## Abstract

**Background:**

Immunization is essential to prevent between 2 and 3 million deaths globally each year and it is widely accepted that it is one of the most cost-effective health interventions. Despite all its advantages, immunization in Ethiopia is still far from the target set by the United Nations Sustainable Development Goals to achieve universal immunization by all countries in 2030. The 2016 Ethiopian Demographic and Health Survey (EDHS) reported an overall full immunization rate of only 38.3%. The objective of this study was to evaluate the spatial distribution of under immunization in 12 to 23 months old children and further identify the determinants of under immunization clustering in the Butajira Health and Demographic Surveillance Site (HDSS).

**Methods:**

We conducted a community based sectional survey from March to April, 2016 in Butajira HDSS. We collected data on immunization status from a total of 482 children between the age of 12 to 23 months. We randomly selected household and interviewed mothers and /or observed vaccination cards when available to collect data on child’s immunization status. We also collected the geographic location of all villages within the ten Kebeles using a Handheld Global Positioning System (GPS) (Garmin GPSMAP®). We analyzed the spatial distribution of under immunization and clustering using the SatScan® software which employs a purely spatial Bernoulli’s model. We also ran a logistic regression model to help evaluate the causes of clustering.

**Results:**

We found that only 22.4% [95% CI: 18.9, 26.4%] of children were fully immunized. This study identified one significant cluster of under immunization among children 12–23 months of age within the Butajira HDSS (relative risk (RR) = 1.24,*P* < 0·01). We found that children residing in this cluster had more than 1.24 times risk of under immunization compared with children residing outside of the identified cluster. We found significant differences with regard to Maternal Tetanus Toxoid immunization status and place of delivery between cases found within a spatial cluster and cases found outside the cluster. For example, the odds of home delivery is more than two times [AOR 2.21: 95%CI; 1.06, 4.63] among children within an identified spatial cluster than the odds among children found outside the identified cluster.

**Conclusions:**

Under immunization of 12–23 months old children and under immunization with specific vaccines such as Polio, BCG, DPT (1–3) and Measles clustered geographically. Spatial studies could be effective in identifying geographic areas of under immunization for targeted intervention like in this study to gear health education to the specific locality.

**Supplementary Information:**

The online version contains supplementary material available at 10.1186/s12887-021-02690-4.

## Introduction

According to the center for disease control and prevention (CDC), Vaccination is defined as the act of introducing a vaccine into the human body to produce immunity to a specific disease [1]. And the World Health Organization (WHO), reports that immunization helps to prevent between 2 and 3 million deaths globally each year [2]. WHO set out a plan which aimed to achieve a world free from vaccine preventable diseases by the year 2020 by envisioning vaccination coverage of greater than 90% nationally and greater than 80% in every district [3]. It also stated that children are considered Fully vaccinated when they have received a BCG vaccination against tuberculosis, three doses each of the DPT and polio vaccines, and a measles vaccination by the age of 12 months.

Currently the Ministry of Health (FMOH) has set ambitious targets [4] to achieve above 95% coverage of Pentavalent 3 and Measles vaccination and also it has set a goal of nationally eradicating polio and measles by the year 2020.

Recent figures for the Ethiopian national immunization coverage level in 2013 and 2014 showed that immunization coverage rose to 87 and 83% coverage, respectively [4], for Pentavalent 3 and Measles vaccination, but still the EDHS 2011 report [5] showed that the overall country level Full immunization is relatively low at 24% with a similar 24% Full immunization coverage for the SNNPR.

In addition, different literatures report varied figures of full Immunization coverage [6–11] in Ethiopia which is sometimes as high as 76% and as low as 36% showing marked Regional and Sub- Regional variations.

Ethiopia launched the expanded programme on immunization in 1980 with the objective of increasing the coverage by 10% annually thus making a significant progress. During the 1990’s implementation of Universal Child Immunization (UCI) initiative made further gains. Then came the so called reaching every district (RED) approach which began implementation since 2004 in districts with poor immunization coverage and high dropout rates and it resulted in a significant improvement. However, the variation in coverage remained among regions. Currently, the RED strategic approach is recast to reaching every children/community strategic approach in order to deal with inequities within districts [12].

There are different articles depicting the utilization of spatial techniques to visualize geographic distributions of different diseases [13–24] like malaria and leshmaniasis but we were only able to find only one literature conducted on child under immunization clustering [25], which was done in USA in 2015 using the spatial scan statistic.

In spite of the above varied prevalence of under immunization there has been lack of studies showing the geographical distribution of clusters of under immunization and cluster specific determinants of under immunization in Ethiopia, so with this fact in mind this study was set out to study the spatial distribution of under immunization and predictors of geographic clustering of under immunization cases among children aged 12 to 23 months old in Butajira HDSS, Southern Ethiopia .

## Methods

### Study setting

The study area was Butajira HDSS owned and administered by the School of Public Health, Addis Ababa University.

The Butajira HDSS site includes 10 *Kebeles* (*kebeles*; a *kebele* is the smallest administrative unit in Ethiopia), which are located in two zones (zones; a zone is an administrative unit in Ethiopia consisting of *Woredas*) in the Southern Nations Nationalities and Peoples Region (SNNPR) [25]. There are three *Woredas* (*Woredas*; a *Woreda* is an administrative unit in Ethiopia consisting of *kebeles*) including, Meskan Woreda and Mareko Woreda from Guraghe Zone and one *Woreda* from Silte zone the Town of Butajira, which is the capital of Meskan *Woreda*. There were about 14,000 Households in the Butajira HDSS at the time of this study.

“Meskan” *Woreda* includes six *Kebeles* namely “Dirama”, “Shershera Bido”, “Bati Lejano”, “Dobena”, “Misrak Meskan” and “Wurib”, whereas “Mareko” *Woreda* consists of “Hope Jale Demeka” and “Mekakelegna Jale Demeka” *Kebeles*. “Dobena” and “Yeteker” are the other two *Kebeles* from Silte Zone and the Tenth *Kebele* is Kebele 04 from Butajira Town [26].

The HDSS population was estimated at 76,350 in 2015. Of the Ten sites nine of them were Rural and one is Urban located in Town of Butajira. Guraghe is the main ethnic group, which is further divided into minor ethnic groups or tribes like Meskan, Mareko, Sodo, Silti and Dobi. Islam is the main religion having two-thirds of the people following the religion: while Orthodox Christianity is the second dominant religion in the area. Guragigna is the major language. Amharic, the national language, is also widely spoken in the area, and is an important written language [26].

### Population

The study population were those children aged 12–23 months in Butajira HDSS which have been selected by simple random sampling from each Kebele using a sample frame of all children 12 to 23 months old during the data collection period, provided from the Butajira Rural Health Project coordinating office located in the School of Public Health, Addis Ababa University.

### Study design

We employed a community based cross-sectional study to evaluate the spatial distribution of under immunization status of 12–23 months old children in Butajira HDSS.

### Sample size

The sample size for this study was determined using single proportion formula [27] and the following assumptions, Prevalence of Full immunization in SNNPR 24% [5], Margin of error of 4% and Confidence interval of 95%. By using EpiInfo version 7, using the single population formula $$ \left[\ n=\raisebox{1ex}{${Z_{\raisebox{1ex}{$\alpha $}\!\left/ \!\raisebox{-1ex}{$2$}\right.}}^2\left(p\ast \left(1-p\right)\right)$}\!\left/ \!\raisebox{-1ex}{${d}^2\ $}\right.\right] $$, the sample size was determined to be 438 and after adjusting for 10% non response rate, the final sample size was determined to be 482 households.

### Sampling technique

The HDSS was stratified into Ten *Kebeles,* then proportional to size samples [27] were selected from the Ten *Kebeles* based on the number of Households per *Kebeles* which have been pre-selected according to the Household number using simple random sampling from the sampling frame provided by the Butajira Rural Health Project coordination office in the School of Public Health, Addis Ababa University. If the Household selected did not have 12 to 23 months old children the next nearby household along the direction of household numbering was considered. If there was more than one child per household all were included in the study. A household is typically defined as a family unit with a single land owner.

### Variables

The independent variables in this study were characteristics of the mother including, Education level, Wealth status, Marital status, Religion, Age of mother, Residence, Occupation and Health service utilization by the mother, Mothers knowledge about VPD and child Immunization and Geographical coordinates of the Household in Latitude and Longitude. The Dependent variable in this study was Under Immunization clustering of children 12 to 23 months old.

### Measurements

The geographic location of all villages / “Gotes” within the ten Kebeles was determined using a Handheld Global Positioning System (GPS) (Garmin GPSMAP®) having an accuracy of + 10 m [28].

### Data collection and quality control

In order to have a quality data a pretest was done by the enumerators of the Butajira HDSS outside of the HDSS site in the Butajira town, before the actual data collection process commenced. Some minor changes in the remarks session was made after the feedback obtained during the pre testing of the questionnaire by the data collectors. Data was then collected by twenty data collectors who are permanent enumerators of the Butajira HDSS, who were trained two times, one before pre testing and another after pre testing. The training covered issues like confidentiality, ethical issues and also the proper way of completing the questionnaire. The data collection technique used for this study was interview of mothers using a structured, pretested questionnaire to measure immunization status by combining methods like interviewing the mother, observation of the households and checking or analysis of the vaccination card kept at home if it was available at the time of interview. The data collection questionnaire used for collecting immunization status from vaccination card was adopted from the 2011 EDHS [5] whereas the rest of the tool was was developed by the investigators of this research work [29]. The data collection period was a week from March 25 to April 1, 2016. Data quality was kept by daily checking completed questionnaires for completeness of data and by both the principal investigator and two experienced supervisors of the Butajira HDSS during the data collection period.

### Data entry and analysis

EpiData Version 3.1 was used for the data entry and Stata 13.0 (StataCorp, College Station, TX) for cleaning and analysis. Each record was given a unique id starting from 1 and running up to 482, and the same unique id was recorded in each questionnaire for the purpose of future reference during data cleaning. SaTScan™ v9.4.2 software (http://www.satscan.org) was used to identify locations and estimate cluster sizes.

A principal component analysis [30] was applied in order to construct a relative household wealth index. The variables included in the principal component analysis model were ownership of land, type of house and construction materials used, availability of fixed assets such as a radio, television, telephone, bed, chair and other household items, possession of domestic animals, time it takes for fetching water for drinking and cooking, and availability and type of latrine. Then a relative socio-economic status was determined by dividing the resulting score into quintiles, with the top 20% being “Richest” and the bottom 20% being “Poorest”.

In our study, the dependent variable was under immunization clustering among 12 to 23 months old children.

According to the WHO guideline [1], “Complete or Full immunization” coverage is defined as a child who has received a BCG vaccination against tuberculosis; at least three doses of DPT vaccine to prevent Diphtheria, Pertusis, and Tetanus (DPT); at least three doses of polio vaccine; and one dose of measles vaccine. We recoded each variable into 0 and 1. No responses were recoded as “0” and labeled “No”. Households with no children between 12 to 23 months old were replaced with the nearby household having a child with the defined age range.

Data about immunization was measured by information from the vaccination card if the mother presented the vaccination card at the time of questioning or by mothers recall if the mother didn’t present the vaccination card. From the vaccination card, all the Yes - No scores were added for each vaccine and were set to give Immunization status of “yes” and coded as “1”, if the score from the vaccination card for the vaccines BCG, polio (1–3),DPT (1–3) and Measles summed up to 8 or greater than 8,where as if the score from the vaccination card for the vaccines BCG, Polio (0–3),DPT (1–3) and measles summed up to be 7 or less they were labeled as “No” and coded as “0”in the “Immunization status”. The immunization status was recoded as “1” if the child had received all the doses of vaccinations and categorized as “Fully immunized” or “0” if the child had missed one or more doses of the vaccinations and categorized as “Under immunized”. Regarding immunization status from mothers recall the reported frequency of child vaccination for the vaccines of BCG, polio (1–3),DPT (1–3) and measles were recorded and summed up and if the sum was determined to be 8 the child was given an immunization status of “Fully immunized” and coded as “1”, on the other side if the sum was determined to be 7 or less the child was given an immunization status of “Under immunized” and coded as “0”.

### Analysis of spatial clustering

The SaTScan™ v 9.4.2 software (http://www.satscan.org) was used to identify clusters of under immunization in Butajira HDSS. The spatial scan statistics, developed by Martin Kulldorf [31] employing a circular scanning window; in this case the Bernoulli’s model was employed. These windows were centered on the 32 geographical coordinates (Latitudes and Longtiudes) of the villages/ “*Gotes*” within the Ten *Kebeles* in the Butajira HDSS and the window size varied from 0 to 50% of the study population, which allowed for both small and large clusters to be detected. A likelihood ratio test was used by the spatial scan statistics to check if the distribution of under immunization cases is random (Null Hypothesis) or occurs in a cluster (the actual number of cases is greater than the expected number of cases) at some areas .

### Analysis of the determinants of clustering

Logistic regression [32, 33] was used to identify the determinants of under immunization clustering by comparing cases of under immunized children identified within the spatial cluster with those cases outside the cluster. This was done by taking the unique ID of under immunized children determined to be within the under immunization cluster from the SaTScan™ v9.4.2 software output window and they were coded as “1” for the dependent variable of “under immunization cluster” and those under immunized children not included in the under immunization cluster were coded as “0” for the the variable “under immunization cluster”. Bivariate logistic regression was then run and those predictor variables with a *P* value of less than 0.2 were considered for the multivariable analysis of determinants of under immunization clustering. Before running the multivariable regression a multi-collinearity test was done. All the regression was done by using Stata 13.0 (StataCorp, College Station, TX) software.

## Results

A total of 482 mothers of children aged between 12 and 23 months old were interviewed, with a response rate of 100%.

### Characteristics of the respondents

The majority of mothers, 470 (97.5%) were married and 273 (77.3%) of the mothers were housewives in occupation. Almost half (48.7%) of the study population belonged to.

Meskan ethnic group, while Silte (19.9%) and Sodo (7.9%) were others (Table 1).

**Table 1:** Socio–demographic characteristics of mothers in Butajira HDSS, Southern Ethiopia, April, 2016 (*n* = 482)

Variable	Frequency	Percent (%)
Educational status		
Did not attend school	268	55.6
Attended School	214	44.4
**Primary**	136	63.5
**Secondary**	57	26.5
**Certificate and Higher**	21	9.8
Marital status		
**Married**	470	97.5
**Divorced**	3	0.62
**Separated or Widowed**	6	1.25
**Single**	3	0.62
Main Occupation		
**House wife**	273	77.3
**Merchant**	88	18.2
**Other (Government employee, Farmer or Daily Laborer)**	21	4.2
Religion		
**Muslim**	377	78.2
**Orthodox Christian**	59	12.2
**Protestant**	44	9.13
**Other**	2	0.14
Ethnic group		
**Welene**	8	1.6
**Sodo**	38	7.9
**Dobi**	13	2.7
**Meskan**	235	48.7
**Mareko**	53	11
**Silti**	96	19.9
**Other**	39	8.1
Mothers age in years		
**15–24**	82	17
**25–34**	314	65
**35–49**	86	18
Number of births by the Mother		
**1**	89	18.5
**2–5**	282	58.5
**> 6**	111	23

The mean age of the mothers was 29.6 (SD = 5.17) years, ranging from 17 to 43 years (Table 1). Only 136 (28.2%) and 78(16.2%) of the mothers attended primary education and secondary or higher level of education, respectively (Table 1).

### Immunization coverage

From the total 484 children of 12–23 months old children included in the study only 22.4, 95% CI (18.9, 26.4%) were Fully immunized while the rest 77.6, 95% CI (73.6, 81.1%) were under immunized.

### Geographic clusters of under immunization

**Fig. 1:**
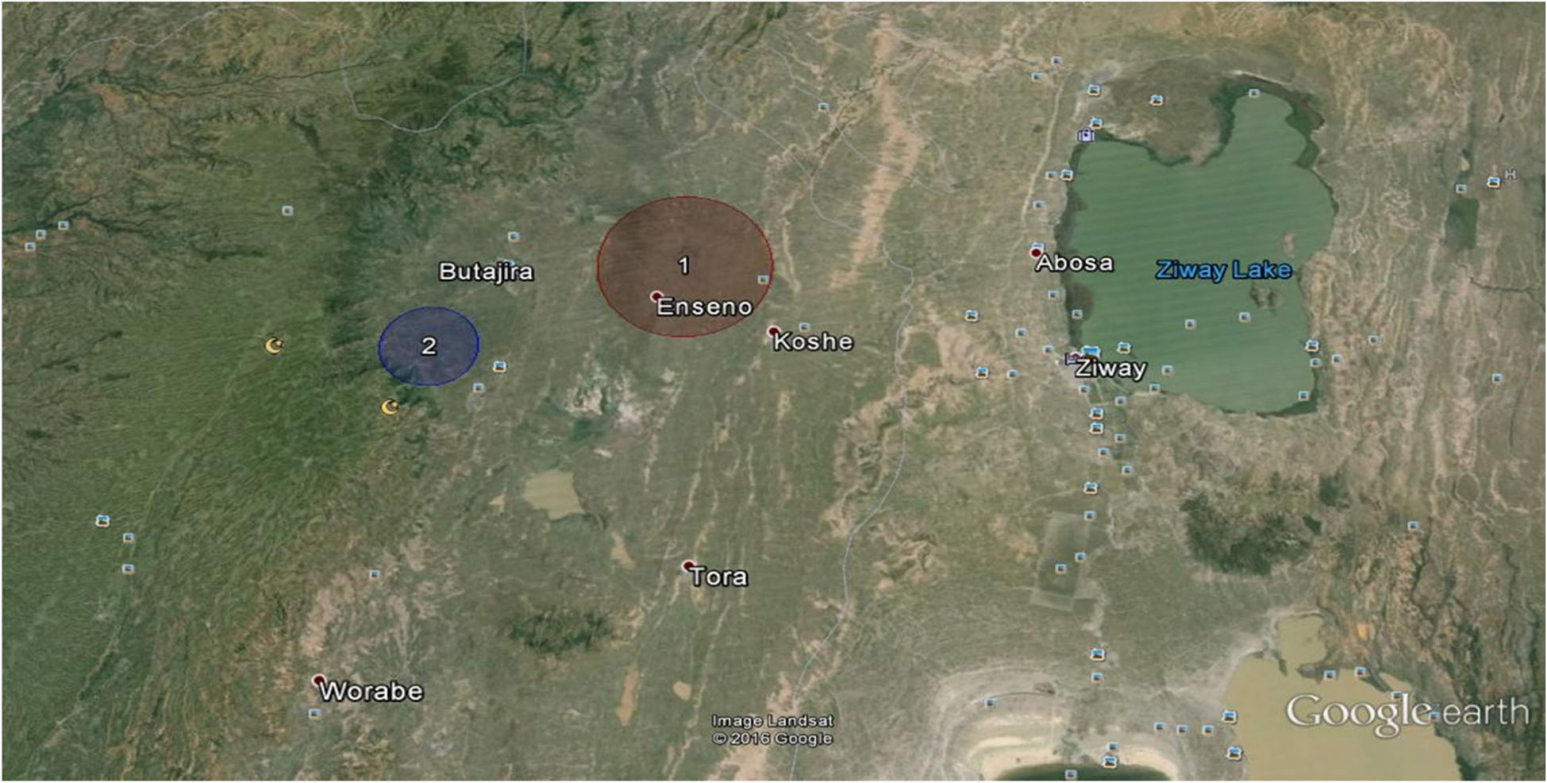
Clusters of under immunization, Butajira HDSS, Southern Ethiopia, April, 2016(*n* = 375), (Green circle is area of high rate of under immunization: Blue circle is area of low rate of under immunization)

The spatial analysis for the period (March to April, 2016) identified one statistically significant cluster of under immunized children of 12 to 23 months old in Butajira HDSS (Fig. 1).

The most statistically significant overall under immunization cluster covered an area East of Butajira town (Fig. 1), and includes part of the Kebeles of Bati Lejano to the north and Mekakelegna Jare demeka to the south. Children in this cluster had 1.24 (*P* value = 0.011) times the rate of under immunization compared with children outside of that cluster (Table 2).

**Table 2:** Spatial Cluster of children under immunization, Butajira HDSS, Southern Ethiopia, April, 2016

Cluster Number and General Description	Radius, km	Population in Cluster, n	Under immunized Children in Cluster, %	Expected Under immunized Cases, n	Actual Under immunized Cases, n	RR	***P*** Value
1:Under immunization cluster, Enseno Town	6.55 km	80	92.5	61.98	74	1.24	0.011*
1.Polio cluster	< 1 km	24	70.8	3.14	17	7.05	< 0.001 ***
2.Polio cluster	0.34 km	35	37.1	4.57	13	3.32	0.0052**
1.BCG cluster	< 1 km	24	70.8	3.14	17	7.05	< 0.001 ***
2. BCG cluster	0.34 km	35	37.1	4.57	13	3.32	0.0052**
1. DPT Cluster, Enseno	8.44 km	104	88.5	58.69	92	1.86	< 0.001 ***
2. DPT Cluster	1.46 km	43	97.7	24.27	42	1.86	< 0.001 ***
3. DPT Cluster	< 1 km	24	91.7	13.54	22	1.68	0.010*
4. DPT Cluster	0.93 km	42	81	23.7	34	1.5	0.027*
5. DPT Cluster	2.96 km	28	85.7	15.8	24	1.57	0.029*
1.Measles cluster,	< 1 km	24	75	5.33	18	3.86	< 0.001 ***
2.Measles cluster, Enseno	2.96	28	64.3	6.22	18	3.28	< 0.001 ***

* *P* Value statistically significant below 0.05

** *P* Value statistically significant below 0.01

*** *P* Value statistically significant below 0.001

**Fig. 2:**
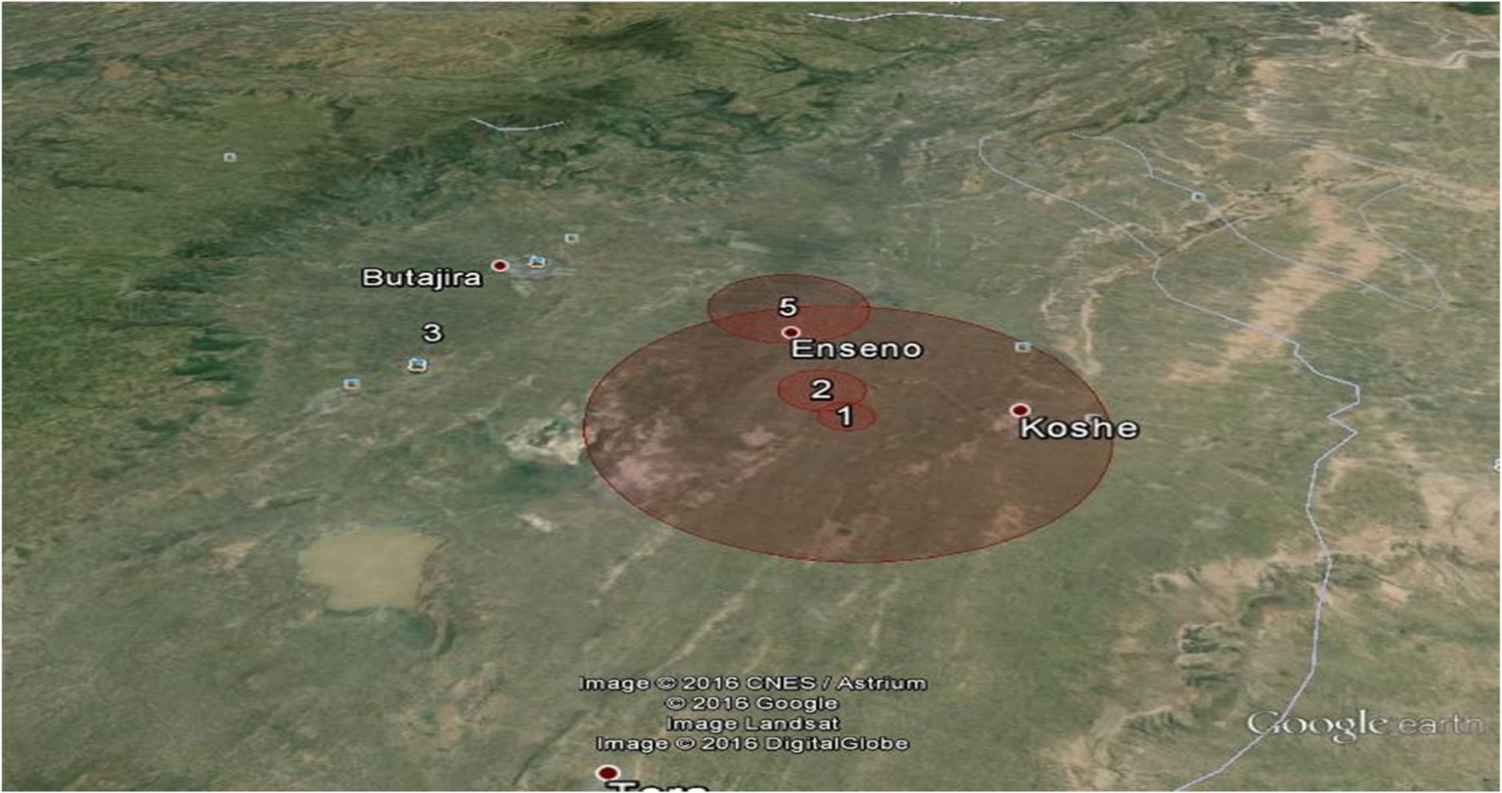
Clusters of DPT (1–3) under immunization, Butajira HDSS, Southern Ethiopia, April, 2016(*n* = 272)

**Fig. 3:**
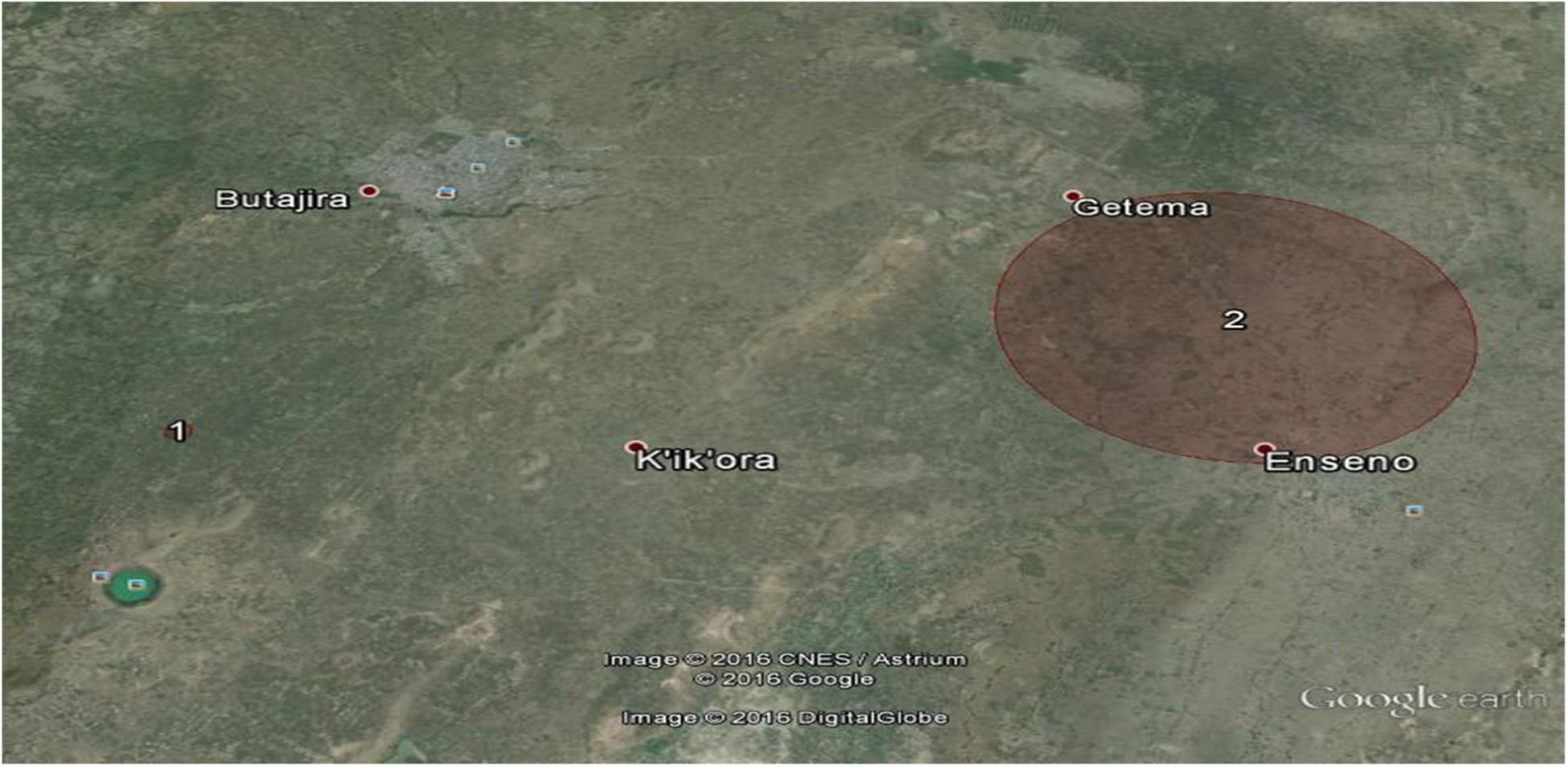
Clusters of Measles under immunization, Butajira HDSS, Southern Ethiopia, April, 2016(*n* = 107)

Overall our study identified 12 significant clusters of under immunization one being the overall under immunization cluster with the six standard antigens and the other 11 being clusters of individual vaccines under immunization (Fig. 2, Fig. 3). The largest clusters were DPT cluster 1 and the overall under immunization cluster with a radius of 8.44 km and 6.55 km, respectively with both clusters located to the east of Butajira town around Enseno town (Fig. 2).

Of the 484 children in the study population evaluated from March to April, 2016, 80 (16.59%) were identified as being within the cluster of under immunization. The under immunization rate was 92.4% within the clusters, compared with 74.69% outside them (Table 2).

### Multivariable analysis of under immunization clustering

After determining clusters of under immunization, determinants of under immunization clustering were evaluated by using logistic regression, a binary logistic regression was run and variables producing a *P*-value > 0.25 were considered as for the multivariable logistic regression (Table 3). Before running the Multivariable logistic regression a multi-collinearity test was done and the result showed no or little collinearity evidenced by variance inflation factors (VIF) and tolerance values of very close to 1(one). The multivariable logistic regression model was also checked for goodness of fit by using the Hosmer-Lemeshow test [32] and it showed that the model was a good fit for the data with a *P* value of 0.0561.

**Table 3:** Multivariable analysis of Cluster of Under immunization of children 12 to 23 months old, Butajira HDSS, Southern Ethiopia, April, 2016 (*n* = 375)

Variables	COR with 95% CI	AOR with 95% CI, ***P*** Value
**Wealth index**	*P* value = 0.0466	
Richest	1.00	
Poor	0.44 [0.22,0.87]	0.83 [0.37,1.88], *P* = 0.661
Middle	0.66 [0.3,1.46]	0.59 [0.25,1.456], *P* = 0.257
Rich	1.0 [0.49, 2.06]	0.93 [0.41, 2.13], *P* = 0.87
**School attendance**	*P* value = 0.0032	
Yes	1.00	
No	2.24 [1.29, 3.9]	1.34 [0.69, 2.58], *P* = 0.39
**Mothers main occupation**	*P* value = 0.1891	
Merchant	1.00	
Housewife	2.06 [0.89,4.75]	1.87 [075, 4.69], *P* = 0.181
Others (Gov.Emp, Farmer, Laborer)	2.06 [0.53,7.98]	1.97 [0.43, 9.08], *P* = 0.387
**Knowledge about VPD**	*P* value = 0.0725	
Yes	1.00	
No	0.41 [0.14, 1.2]	0.97 [0.26,3.56], *P* = 0.963
**Source of information about VPD**	*P* value = 0.0417	
Health professionals	1.00	
Radio/Television	0.42 [0.18,0.98]	0.62 [0.25,1.55], *P* = 0.308
Neighbors or Friends/school	0.49 [0.2, 1.23]	0.77 [0.28,2.08], *P* = 0.604
**Mothers TT vaccination**	*P* value = 0.0084	
No	1.00	
Yes	2.2 [1.19, 4.06]	**2.21 [1.06,4.63],** ***P*** = 0.035*
**Availability of child vaccination card at home**	*P* value = 0.0002	
Yes	1.00	
No	3.54 [1.69, 7.39]	2.15 [0.95, 4.88], *P* = 0.066
**Place of birth of last pregnancy**	*P* value = 0.0000	
Health care setup (Hospital, Health center or Health post)	1.00	
Home or TBA	6.43 [3.48, 11.88]	**5.1 [2.56,10.1],** ***P*** < 0.001***

* *P* Value statistically significant below 0.05

** *P* Value statistically significant below 0.01

*** *P* Value statistically significant below 0.001

The multivariable analysis of the determinants of under immunization clustering in Butajira HDSS revealed that mothers who gave birth in their homes or with a TBA for the last pregnancy were 5.1 times at greater odds of being within the cluster of under immunization than those mothers who gave birth in a hospital, health center or health post during their last pregnancy (Table 3).

Another significantly associated factor with under immunization clustering within the Butajira HDSS was having received TT vaccination by the mothers who were 2.21 times at greater odds of being in the cluster than those mothers who didn’t receive TT vaccination (Table 3).

## Discussion and conclusions

In this paper we evaluated the spatial clustering of under immunization among 12–23 months old children in a demographic surveillance area. We found that the spatial distribution of under immunization is not randomly distributed, rather it exhibited a spatial clustering. Moreover, we found that children who are found in the identified spatial cluster are different from those children who are found out of the spatial cluster with respect to place of delivery and mother’s immunization status (Table 3).

The present study identified statistically significant under immunization clusters of varying size in different locations within the DSS site. The most significant cluster was found in the lowland part of the Butajira HDSS (Fig. 1). A Similar study reported little area level clustering of under immunization in Kaiser Permanente, Northern California,USA [25]. Using a SatScan® we determined the size and location of cluster of under immunization in the district. Children living in this cluster had a higher risk for under immunization than the expected risk for the underlying population.

We further evaluated the cause of the clustering of under immunization and found out that giving birth at home or with TBA was the major predictor of under immunization clustering, this could be explained due to the fact that those who deliver at health institutions are given advice about child immunization which affects their practice positively, since institutional delivery is associated with increased health service utilization,whereas those mothers who gave birth at home or by a TBA lack such advice and so are more likely to under immunize their child [34–36].

Also maternal TT vaccination was found to be a predictor of under immunization cluster which may be possibly explained by the common understanding that mothers may perceive their children as protected by the Maternal TT vaccination and also the pain associated with the vaccine injection could make the mothers to refrain from child immunization. Another explanation is the increased utilization of Health services like child immunization among mothers giving birth at health institutions [37].

Spatial studies including the current study have been shown to be effective in pinpointing areas of disease clustering, which could be very useful to programme managers of public health institutions in Ethiopia and beyond. Geographically targeting such areas which have high burden of under immunization for under immunization could be a cost effective method to tackle the problem specifically [14].

We believe that the current work has a number of limitations. First, we used village /“*Gote*” level geographic location rather than household GPS. This might have resulted in a reduction in the number of clusters identified in the present report. Secondly we used a circular scanning window which could have missed the actual shape of the clusters that might have had an oval or irregular shape.

## Conclusions

In conclusion the present study identified spatial clusters of overall Under immunization of 12–23 months old children and vaccine specific under immunization with polio, measles, BCG and DPT (1–3) vaccines were observed in the Butajira HDSS. Having received TT vaccination by mother, and delivery of the last pregnancy at home or with a TBA were determined as predictors of under immunization Clustering.

In order to address the problem of under immunization clusters identified in the present study, we recommend that proper health education about benefits of full immunizing children should be planned for the community at large and also at different occasions’ like mothers TT vaccinations.

The health system should be strengthened to increase the access and quality of care of institutional delivery. Further studies are recommended in the area to address the limitations of our study.

## Supplementary Information

**Additional file 1.**

## Data Availability

Data of the research can be obtained upon request from the corresponding author.

## Abbreviations

ANC: Antenatal care
AOR: Adjusted Odds Ratio
BCG: Bacille Calmette-Guérin
CDC: Centers for Disease Control and Prevention
COR: Crude Odds Ratio
DPT: Diphtheria, Pertussis and Tetanus vaccine
EDHS: Ethiopian Demographic Health Survey
EPI: Expanded Programme on Immunization
FMOH: Federal Ministry of Health
HDSS: Health and Demographic Surveillance Site
HepB: Hepatitis B
Hib: *Haemophilus influenzae* B
WHO: World Health Organization

## References

[1] Definition of terms http://www.cdc.gov/vaccines/vac-gen/imz-basics.htm. Acssesed 30 July 2015.

[2] Global Health Observatory (GHO) data [http://www.who.int/gho/immunization/en/]. Acssesed 30 July 2015.

[3] WHO: Immunization. 2015. Acssesed 30 July 2015.

[4] FMOH. Health sector development program IV. Federal democratic republic of Ethiopia ministry of health.[2010/11–2014/15], 2010.

[5] Central Statistical Agency, Ethiopia and ICF International Calverton. Ethiopian Demographic and Health Survey, 2011. Addis Ababa: Central Statistical Agency, ICF International Calverton, Maryland; 2012.

[6] Debie A (2014). Assessment of fully vaccination coverage and associated factors among children aged 12-23 months in Mecha district, North West Ethiopia: a cross-sectional study. Sci J Public Health.

[7] Legesse E, Dechasa W. An assessment of child immunization coverage and its determinants in Sinana District, Southeast Ethiopia. BMC Pediatrics. 2015;15(31). 10.1186/s12887-015-0345-4. 10.1186/s12887-015-0345-4 PMC4438454 25886255

[8] Etana B, Deressa W (2012). Factors associated with complete immunization coverage in children aged 12-23 months in ambo Woreda, Central Ethiopia. BMC Public Health.

[9] Lakew Y, Bekele A, Biadgilign S (2015). Factors influencing full immunization coverage among 12-23 months of age children in Ethiopia: evidence from the national demographic and health survey in 2011. BMC Public Health.

[10] Kassahun MB, Biks GA, Teferra AS. Level of immunization coverage and associated factors among children aged 12–23 months in Lay Armachiho District, North Gondar Zone, Northwest Ethiopia: a community based cross sectional study, BMC Res Notes. 2015;8(239). 10.1186/s13104-015-1192-y. 10.1186/s13104-015-1192-y PMC4467673 26071403

[11] Tadesse H, Deribew A, Woldie M (2009). Predictors of defaulting from completion of child immunization in 0south Ethiopia, may 2008: a case control study. BMC Public Health.

[12] FMOH. Ethiopia National Expanded Programme on immunization, comprehensive multi-year plan 2016–2020. Federal ministry of health, Addis Ababa, Ethiopia: Addis Ababa; 2015.

[13] Guimarães AGF, Alves GBM, Pessoa A de M, da Silva Junior NJ (2015). Spatial analysis of visceral leishmaniasis in the municipality of Rondonópolis, in the Brazilian State of Mato Grosso, from 2003 To 2012: human, canine and vector distribution in areas of disease transmission. Rev Soc Bras Med Trop.

[14] Santos C, Araujo K, Jardim-Botelho A, et al. Diarrhea incidence and intestinal infections among rotavirus vaccinated infants from a poor area in Brazil: a spatial analysis. BMC Public Health. 2014;14(399). 10.1186/1471-2458-14-399. 10.1186/1471-2458-14-399 PMC4047769 24761937

[15] Coleman M, Coleman M, Mabuza AM, Kok G, Coetzee M, Durrheim DN (2009). Using the SaTScan method to detect local malaria clusters for guiding malaria control programmes. Malar J.

[16] Havulinna AS, Pääkkönen R, Karvonen M, Salomaa V (2008). Geographic patterns of incidence of ischemic stroke and acute myocardial infarction in Finland during 1991-2003. Ann Epidemiol.

[17] Havulinna ASTP, Marttila RJ, Martikainen KK, Eriksson JG, Taskinen OME, Karvonen M (2008). Geographical variation of medicated Parkinsonism in Finland during 1995 to 2000. Move Disord.

[18] Huang SSYD, Stelling J, Placzek H, Kulldorff M (2010). Automated detection of infectious disease outbreaks in hospitals: a retrospective cohort study. PLoS Med.

[19] Menezes JA, De Castro Ferreira E, Andrade-Filho JD, De Sousa AM, Morais MHG, Rocha AMS, et al. An integrated approach using spatial analysis to study the risk factors for leishmaniasis in area of recent transmission. BioMed Research International; 2015. 10.1155/2015/621854. 10.1155/2015/621854 PMC4502282 26229961

[20] Kikuti M, Cunha GM, Paploski IAD, Kasper AM, Silva MMO, Tavares AS, et al. Spatial distribution of dengue in a brazilian urban slum setting: role of socioeconomic gradient in disease risk. PLoS Negl Trop Dis. 2015;9(7). 10.1371/journal.pntd.0003937. 10.1371/journal.pntd.0003937 PMC4510880 26196686

[21] Kousa A, Havulinna AS, Moltchanova E, Taskinen O, Nikkarinen M, Eriksson J, Karvonen M (2006). Calcium:magnesium ratio in local groundwater and incidence of acute myocardial infarction among males in rural Finland. Environ Health Perspect.

[22] Peterson I, Borrell LN, El-Sadr W, Teklehaimanot A (2009). A temporal-spatial analysis of malaria transmission in Adama, Ethiopia. Am J Trop Med Hyg.

[23] O’Meara W, Smith N, Ekal E, Cole D, Ndege S. Spatial distribution of bednet coverage under routine distribution through the publichealth sector in a rural district in Kenya. PLoS One. 2011;6(10). 10.1371/journal.pone.0025949. 10.1371/journal.pone.0025949 PMC3192112 22022481

[24] Gebreyesus S, Damen HM, Woldehanna T, Lindtjørn B. Local spatial clustering of stunting and wasting among children under the age of 5 years: implications for intervention strategies. Public Health Nutr. 2015:1–11. 10.1017/S1368980015003377. 10.1017/S1368980015003377 PMC10270919 26700548

[25] Lieu T, Ray T, Klein N, Chung C, Kulldorff M. Geographic Clusters in Underimmunization and Vaccine Refusal. Pediatrics. 2015;135(2). 10.1542/peds.2014-2715. 10.1542/peds.2014-2715 25601971

[26] Berhane Y, Byass P. Butajira DSS Ethiopia. In: INDEPTH Network, ed. population and health in developing countries. Volume 1. Part III. INDEPTH DSS site profiles: International Development Research Centre; 2002. (Available from: http://www.idrc.ca/en/ev-9435-201-1-DO_TOPIC.html#begining. Accessed 20 Dec 2015.

[27] Cochran WG. Sampling survey techniques. North Carolina State University. Dept. of Statistics; 1948.

[28] GPS Accuracy | Garmin Support. Available from: https://support.garmin.com/en-US/?faq=aZc8RezeAb9LjCDpJplTY7. Accessed 19 Feb 2021.

[29] Ketsela A, Hagos S, Deressa W. English version questionnaire. Spatial distribution of under immunization among 12–23 months old children in Butajira HDSS; 2016.

[30] Jolliffe IT (1986). Principal components in regression analysis. Principal component analysis.

[31] Kulldorff M (1997). A spatial scan statistic. Commun Stat - Theory Methods.

[32] Agresti A. Categorical data analysis: Wiley; 2003.

[33] Hosmer DW Jr, Lemeshow S, Sturdivant RX. Applied logistic regression: Wiley; 2013.

[34] Yaya S, Bishwajit G, Ekholuenetale M. Factors associated with the utilization of institutional delivery services in Bangladesh. PLoS One. 2017;1;12(2). 10.1371/journal.pone.0171573. 10.1371/journal.pone.0171573 PMC5305190 28192478

[35] Amano A, Gebeyehu A, Birhanu Z (2012). Institutional delivery service utilization in Munisa Woreda , South East Ethiopia: a community based cross-sectional study. BMC Pregnancy Childbirth.

[36] Bayu H, Fisseha G, Mulat A, Yitayih G, Wolday M (2015). Missed opportunities for institutional delivery and associated factors among urban resident pregnant women in South Tigray zone, Ethiopia: a community-based follow-up study. Global Health Action.

[37] Biks GA, Tariku A, Tessema GA. Effects of antenatal care and institutional delivery on exclusive breastfeeding practice in Northwest Ethiopia: a nested case – control study. Int Breastfeed J. 2015:1–6. Available from:. 10.1186/s13006-015-0055-4. 10.1186/s13006-015-0055-4 PMC4653867 26594231

