# Supplementary material for: Spatial distribution of under immunization among children 12–23 months old in Butajira HDSS, southern Ethiopia
Source: BMC Pediatr. 2021 May 10;21:226. doi: 10.1186/s12887-021-02690-4 (PMC8108332; doi:10.1186/s12887-021-02690-4)
Supplement: Supplementary file 1 — Additional file 1. [file 12887_2021_2690_MOESM1_ESM.docx]

**English Version Questionnaire**

**Research title : Spatial distribution of Under-Immunization among children 12-23 months old in Butajira HDSS , Southern Ethiopia**

Admassu Ketsela Mengistu * (B.Pharm, MPH)^1^, Seifu Hagos Gebreyesus (PhD)^2^ , Wakgari Deressa (PhD)^3^

1. Menelik II Medical & Health Science College, Kotebe Metropolitan University, Addis Ababa, Ethiopia

2. Department of Nutrition and Dietetics , School of Public Health, Addis Ababa University, Addis Ababa, Ethiopia

3. Department of Preventive Medicine, School of Public Health, Addis Ababa University, Addis Ababa, Ethiopia

***** corresponding author (admeketsela@gmail.com)

**English version questionnaire**

| **Research title : Spatial distribution of Under-Immunization in Butajira District** | | |
| --- | --- | --- |
| *This questionnaire form is to be used at Households to record information on the vaccinations for children aged 12 to 23 months.* | | |
| **IDENTIFICATION** | | |
| **1**. Zone Name :___________________ | | **2**. Kebele/Village Name:____________________ |
| **3**. Household number: ___________________ 4 . Sex of the child ________________________ | | |
| 5. Household GPS Coordinate | |  |
| **N** | **E** |  |

**SECTION I: Sociodemographic Characterstics**

| **S.No** | **QUESTION** | | **CODING** | | **REMARK** |
| --- | --- | --- | --- | --- | --- |
| 101 | What is your Marital status ? | | Married.............1  Divorced...........2  Separated..........3  Widowed...........4  Single................5 | |  |
| 102 | How old are you ? | | __________Years | |  |
| 103 | Have you ever attended school ? | | Yes……………1  NO………….…2 | | If NO Go To Qn.105 |
| 104 | What is the highest Education level you achieved ? | | Primary(1-6).......................1  Secondary(7-10).................2  Certificate............................3  Degree and above ............4 | |  |
| 105 | What is your Main Occupation? | | Housewife...............................1  Government employee...........2  Merchant.................................3  Farming /animal husbandry....4  Daily Labourer........................5  Others, Specify_____________ | |  |
| 106 | What is your Religion? | | Muslim..........................1  Orthodox........................2  Protestant.......................3  Other, specify _­­­­­________ | |  |
| 107 | What is your Ethnicity? | | Welen..........................1  Sodo.............................2  Dobi.............................3  Meskan........................4  Mareko........................5  Silti .............................6  Other (specify)________ | |  |
| 108 | WHICH OF THIS MATERIALS EXIST IN YOUR HOUSE  YES NO YES NO  Has electricity Has motorcycle  Kerosene lamp Has car  Has radio Has mobile phone  Has television Has landline phone  Has refrigerator Chair  Has bicycle Table  Solar light | | | | |
| 109 | MAIN MATERIAL OF THE FLOOR (Record observation) | Earth/sand ..................................1 Palm/bamboo...............................2  Wood............................................3  Cement ........................................4  Ceramic tiles................................5 Carpet...........................................6  Other(specify............................ | | |  |
| 110 | MAIN MATERIAL OF THE ROOF(record your observation) | Has corrugate iron roofing ...........1  Hatch/leaf/ mud............................2  Has roofing tiles............................3  Has other roofing ..........................4 | | |  |
| 111 | MAIN MATERIAL OF THE WALLS (record observation.) | Nowalls ..........................................1  Rudimentary walls...........................2 Stone with mud ..............................3 Plywood...........................................4  Stone with lime/ cement...................5  Mud and wood..................................6  Cement block..................................7  Other ( specify )--------------------------- | | |  |
| 112 | How long does it take you to fetch water ? | Minutes ...................1 /-------/--------/  Don’t Know.............2 | |  | |
| 113 | What is the toilet facility available ? | Functional flush toilet..................1  Unfunctional flush toilet..............2  Functional pit latrine....................3  Unfunctional pit latrine................4  Forest or open area.......................5  Other( specify ).............................6 | |  | |
| 114 | Main fuel type used for cooking | Electric.......................................1  Solar power...............................2  Kerosene....................................3  Charcoal.....................................4  Wood..........................................5  Leaves........................................6  Cow Dung...................................7  Other( specify )...........................8 | |  | |
| 115 | Do you have agriculture land ? | Yes………………………....…1  NO………………………….…2 | | If No Skip to Question 117 | |
| 116 | How many acres of agricultural land do you own? | Number ...................Acres /-------/---- | | |  |
| 117 | Do you own any livestock? | Yes………………………....…1  NO………………………….…2 | | | Skip to Section II |
| 118 | How many of the following do you own ? | Cow/Ox………….........1 /------/-----/  Horse/Donkey/Mule......2 /------/-----/  Goat...............................3 /------/-----/  Sheep.............................4 /------/-----/  Hen................................5 /------/-----/  Bee Hive........................6 /------/-----/ | | |  |

| **SECTION II: Health Service Utilization** | | | |
| --- | --- | --- | --- |
| **S.No** | **QUESTION** | **CODING** | **REMARK** |
| 201 | Is there any health facility rendering vaccination service near your home? | Yes...........................1  No.............................2 | If NO go to Qn. 203 |
| 202 | If Yes to the above question what type of health facility is it ? | Hospital...................1  Health center............2  Health post................3  Private clinic............4 |  |
| 203 | What was your Place of Delivery for the last pregnancy? | Home/TBA..................1  Health Facility.............2 |  |
| 204 | Have you attended ANC Service during your last pregnancy ? | Yes..................1  No...................2 |  |
| 205 | Have you received TT vaccination during your last pregnancy? | Yes..................1  No....................2 | If NO go to Qn. 207 |
| 206 | If Yes to the above question. How many injections did you receive through your pregnancy? | _______ |  |
| 207 | How many children did you ever gave birth to? | ________ |  |

| **SECTION III: Knowledge And Attitude Towards Immunization** | | | |
| --- | --- | --- | --- |
| 301 | Have you heard about vaccination and vaccine preventable diseases? | Yes..................1  No...................2 | If NO go to Qn.19 |
| 302 | If yes to the above question from which source did you hear about it first ? | Radio...........................1  TV................................2  Friends/Neighbours......3 School..........................4 Health personnel..........5  Others,specify_____ |  |
| 303 | Can you tell me at what age should vaccination be started ? | Right after birth...........1  One month after birth...2 Anytime after birth.......3  After one year..............4  I don’t know.................5 |  |
| 304 | At what age the child should complete Immunization? | _____________ |  |
| 305 | How many vaccination sessions are needed for a child to be fully protected ? | One..............................1  Three...........................2  Five.............................3  Repeated.....................4  Don’t know.................5 |  |
| 306 | Have you ever received a vaccination card for your child ? | Yes..................1  No...................2 | If NO skip to SECTION V QUESTION 50 3 |
| 307 | Do you currently have a vaccination card for your child given from health facility? | Yes..................1  No...................2 | If NO skip to SECTION V QUESTION 50 3 |

**SECTION IV :Immunization Status From Vaccination Card (Adopted from EDHS 2011)**

|  | | | Last Birth | | | | | | Next to Last Birth | | | | | | |  |
| --- | --- | --- | --- | --- | --- | --- | --- | --- | --- | --- | --- | --- | --- | --- | --- | --- |
| Record day, month and year of birth as written on vaccination record | | | __ __ / __ __/ 200 ___ | | | | | | __ __ / __ __ / 200 __ | | | | | | | REMARK |
| Copy dates for each vaccination from the card.Write ‘NO’ in day column if card shows that vaccination was given but no date recorded. | | | Date of Immunization | | | | | | Date of Immunization | | | | | | |  |
|  |  |  |  | | | | | |  | | | | | | |  |
|  |  |  | Day | Month | | Year | | | Day | | Month | | Year | | |  |
| 401 | BCG | BCG |  |  |  |  |  |  |  |  |  |  |  |  |  | IF BCG TO Measles is all recorded skip to section V ,503H and finish |
|  | OPV 0 | POV0 |  |  |  |  |  |  |  |  |  |  |  |  |  |  |
|  | OPV 1 | OPV1 |  |  |  |  |  |  |  |  |  |  |  |  |  |  |
|  | OPV 2 | OPV2 |  |  |  |  |  |  |  |  |  |  |  |  |  |  |
|  | OPV 3 | OPV3 |  |  |  |  |  |  |  |  |  |  |  |  |  |  |
|  | DPT-HepB Hib1 | Penta 1 |  |  |  |  |  |  |  |  |  |  |  |  |  |  |
|  | DPT-HepB Hib2 | Penta 2, |  |  |  |  |  |  |  |  |  |  |  |  |  |  |
|  | DPT-HepB Hib3 | Penta 3 |  |  |  |  |  |  |  |  |  |  |  |  |  |  |
|  | PCV 1 | PCV 1 |  |  |  |  |  |  |  |  |  |  |  |  |  |  |
|  | PCV 2 | PCV2 |  |  |  |  |  |  |  |  |  |  |  |  |  |  |
|  | PCV 3 | PCV3 |  |  |  |  |  |  |  |  |  |  |  |  |  |  |
|  | Rota 1 | Rota 1 |  |  |  |  |  |  |  |  |  |  |  |  |  |  |
|  | Rota 2 | Rota2 |  |  |  |  |  |  |  |  |  |  |  |  |  |  |
|  | Measles | Measles |  |  |  |  |  |  |  |  |  |  |  |  |  |  |

**SECTION V : Immunization Status From Mothers Recall (Adopted from EDHS 2011)**

| 501 | | Has (NAME) received any vaccinations that are not recorded on this card, including vaccinations given in a national immunization day campaign?  RECORD “YES” ONLY IF THE RESPONDENT MENTIONS AT LEAST ONE OF THE VACCINATIONS IN 1 THAT ARE NOT RECORDED AS HAVING BEEN GIVEN | YES-------------1  (PROBE FOR VACCINATIONS SHOWN IN 2AND WRITE “66” IN THE CORRESPONDING DAY COLUMN IN 1 ) (SKIP TO 503)  NO--------------------2  DON'T KNOW-------8 | YES-------------1  (PROBE FOR VACCINATIONS SHOWN IN 2AND WRITE “66” IN THE CORRESPONDING DAY COLUMN IN 1 ) (SKIP TO 503)  NO--------------------2  DON'T KNOW-------8 | ” SKIP TO SECTION VI |
| --- | --- | --- | --- | --- | --- |
| 502 | | Did (NAME) ever have any vaccinations to prevent him/her from getting diseases, including vaccinations received in a national immunization campaign? | YES-------------------1  NO--------------------2  DON'T KNOW------8  (SKIP TO 601) | YES-------------------1  NO--------------------2  DON'T KNOW------8  (SKIP TO 601) |  |
| 503 | | Please tell me if (NAME) had any of the following vaccinations: |  |  |  |
|  | 503A | A BCG vaccination against tuberculosis, that is, an injection in the right arm or shoulders that usually causes a scar? | YES------------------------1  NO--------------------------2  DON'T KNOW-----------8 | YES-------------------1  NO--------------------2  DON'T KNOW------8 |  |
|  | 503B | Polio vaccine, that is, drops in the mouth? | YES---------------------------1  NO----------------------------2  (SKIP TO 503D)  DON'T KNOW--------------8 | YES-------------------1  NO--------------------2  DON'T KNOW------8 | (SKIP TO 503D) |
|  | 503C | Was the first polio vaccine given in the first two weeks after birth or later? | FIRST TWO WEEKS-------1  LATER-----------------------2 | FIRST TWO WEEKS-------1  LATER-----------------------2 |  |
|  | 503D | How many times was the polio vaccine received? | NUMBER OF TIMES---- | NUMBER OF TIMES--- |  |
|  | 503E | A DPT or DPT-HepB-Hib vaccination, that is, an injection given in the thigh or buttocks sometimes at the same time as polio drops? | YES-------------1  NO--------------2 (SKIP TO 503G)  DON'T KNOW--------------8 | YES-------------1  NO--------------2  DON'T KNOW----8 | (SKIP TO 503G) |
|  | 503F | How many times was a DPT or DPT-HepB-Hib vaccination given ? | NUMBER OF TIMES---- | NUMBER OF TIMES---- |  |
|  | 503G | A Measles injection or an MMR injection - that is, a shot in the arm at the age of 9 months or older to prevent him/her from getting Measles? | YES----------------------1  NO-----------------------2  DON'T KNOW---------8 | YES----------------------1  NO-----------------------2  DON'T KNOW---------8 |  |
|  | 503H | Did (NAME) receive a vaccination certificate for completing the schedule for all vaccinations? | YES----------------------1  NO-----------------------2  DON'T KNOW---------8 | YES----------------------1  NO-----------------------2  DON'T KNOW---------8 |  |

**SECTION VI: Reasons for under immunization**

| **S.No** | **QUESTION** | **CODING** | **RE MARK** |
| --- | --- | --- | --- |
| 601 | What are the reasons for the child not to receive full immunization? | Absence of health facility.............................1  Distance of health facility being very far from home............................................................2  Vaccination has no use................................3  Vaccination harms the child.........................4  Religious and cultural reasons......................5  Lack of awareness about vaccination...........6  Vaccination time not convenient..................7 |  |
|  | | | |
| Field editor’s name and signature: | | Main Supervisors name and signature: | |
| Name____________________________ | | Name_______________________ | |
